# Factors associated with self-perceived treatment-resistance in bipolar disorder

**DOI:** 10.1097/MD.0000000000036217

**Published:** 2024-01-05

**Authors:** Toshimasa Fujimura, Daiki Taira, Yoshihiro Uchida, Keitaro Takahashi, Kanako Yamasuji, Kentaro Shimizu, Yasuhito Nagai, Naoto Yoshinari, Tomoe Hirata, Kazuma Fujimoto, Yui Kurosawa, Seita Yasuda, Akane Yoshikawa, Yoshihide Takeshita, Masanobu Ito, Chihiro Kakiuchi, Tadafumi Kato

**Affiliations:** a Department of Psychiatry, Juntendo University School of Medicine, Tokyo, Japan.

**Keywords:** bipolar disorder, real world, self-perceived treatment resistance

## Abstract

Patients with bipolar disorder often report self-perceived treatment resistance. However, it is not known to what extent it is due to actual treatment resistance. The Juntendo University provides “Bipolar Disorder Treatment Rebuilding Program,” in which patients with self-reported treatment resistant bipolar disorder are hospitalized for 2 weeks and undergo detailed examinations. In this study, we report our experience with the initial 43 patients hospitalized during the one and half years after the launch of the program. Among the patients who underwent full assessment, only one was regarded as having genuine treatment-resistant bipolar disorder without comorbidity. In other cases, ten were not diagnosed with bipolar disorder, 3 had organic brain diseases, 12 had comorbid mental disorders and its symptoms were regarded as treatment-resistant bipolar symptoms by the patients, and 18 did not receive adequate treatment because attendant physicians did not adhere to the treatment guidelines or patients did not adhere to the treatment because of lack of insight. The number of participants was not large, and selection bias hampered the generalization of the findings. Insight and adherence were assessed without the use of validated tools. We could not verify recovery after adequate treatment because of the limited hospitalization period. The findings suggest that most patients with self-perceived treatment-resistant bipolar disorder may not have genuine treatment-resistant bipolar disorder. These results shed light on the difficulties of public education of bipolar disorder and importance of providing appropriate services for diagnosis and treatment of bipolar disorder in the community.

## 1. Introduction

Bipolar disorder is a chronic mental disorder characterized by recurrent depressive and (hypo)manic episodes, and is associated with decreased quality of life, caregiver burden, and increased suicidality.^[1]^ There are many effective maintenance treatments, and several treatment guidelines have been established for bipolar disorder.^[2–4]^ Nevertheless, more than one-third of patients with bipolar disorder relapse within one year despite maintenance therapy^[5]^ and more than 90% experience at least one relapse in their lifetime.^[6]^ Rapid cycling and refractory depression often leads to long-term impairment of social functioning, resulting in a large social burden.

Many studies and guidelines have addressed the treatment-resistant bipolar disorder.^[7–11]^ However, the complexity of the clinical presentation, course, and treatment options for bipolar disorder makes it difficult to define treatment resistance.^[8]^ The International Society for Bipolar Disorders Task Force report on terminology related to course and prognosis did not clearly define treatment resistance.^[9]^ The development of definitions and guidelines for intervention in treatment-resistant bipolar disorder has just begun.^[10]^

Treatment-resistant bipolar disorder can be divided into 3 major categories: treatment-resistant depressive episodes, treatment-resistant manic episodes, and difficulty in maintaining remission (including rapid cycling), and definition of treatment resistance would be necessary for each of them.^[10]^ In addition, many definitions of treatment resistance target only pharmacotherapy, and some believe that pharmacotherapy alone is insufficient for the treatment of bipolar disorder. It is also necessary to define multitherapy resistance, which includes psychotherapy/psycho-education and neuromodulation (for example, modified electroconvulsive therapy).^[11]^

However, even considering the above-mentioned factors, the definition of treatment resistance is not straightforward because of the problem of “apparent” and not genuine treatment resistance. Genuine-treatment-resistant bipolar disorder can be defined as a patient not responding to the optimum treatment. However, many patients do not respond well to treatment. The 2020 Collegium Internationale Neuro-Psychopharmacologicum guidelines caution that the following factors should be addressed before labeling a bipolar patient as treatment-resistant: correct diagnosis, disorder not secondary to an organic disorder, poor response to treatment not due to somatic or mental comorbidity, poor response to treatment not due to a somatic condition that might not constitute a disorder by itself, failure of therapy not due to non-tolerability, patient compliance with recommended treatment, and poor response not a consequence of lack of adherence.^[10]^ A certain number of bipolar disorder cases with apparent treatment resistance may be due to these factors.

As discussed above, physicians have tried to define treatment resistant bipolar disorder, and searched for the factors associated with treatment resistance. On the other hand, there are patients who report self-perceived treatment resistant bipolar disorder. However, it is not known to what extent these patients are actually treatment resistant bipolar disorder.

Department of Psychiatry, Juntendo University provides a program named “Bipolar Disorder Treatment Rebuilding Program,” in which patients with self-reported treatment resistant bipolar disorder are hospitalized for 2 weeks and undergo detailed examinations to delineate all the possible factors associated with apparent treatment resistance. In our initial experience with this program for one and half years, we found that most patients with self-perceived treatment-resistant bipolar disorder did not have genuine treatment resistance.

## 2. Method

### 2.1. Subjects

**Figure 1.**
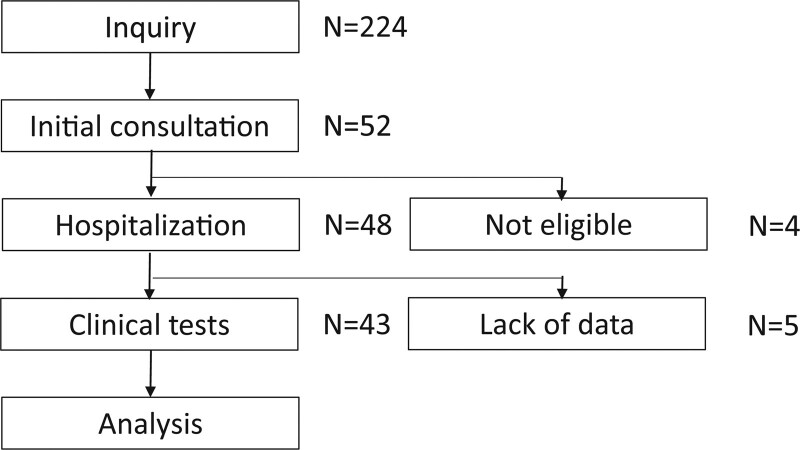
Participants enrolled in the study. Among the 224 participants who contacted the staff of the program, 52 were subject to the initial consultation at the outpatient clinic; 48 met the inclusion criteria and were admitted to the hospital. Of those, 43 completed all the program, whereas 5 dropped out from the program. The data of 43 participants were analyzed in this study.

The bipolar disorder treatment rebuilding program is a two-week hospitalization program for patients with bipolar disorder who report themselves as treatment-resistant. They are intensively scrutinized and medical staff review past treatments from various perspectives. The purpose of the bipolar disorder treatment rebuilding program is announced on the Juntendo University Hospital website. It was announced in the newsletter of Bipolar Disorder Research Network Japan on September 1, 2020, also on corresponding author’s Twitter on September 8, 2020. Furthermore, this program was introduced in a newspaper on December 7, 2021. Patients or their families who want to join this program send an e-mail to a designated address, and staff, that is, a psychologist (D.T.) and psychiatrist (T.K.), check the eligibility of the participants through email. The inclusion criteria are as follows: (1) the patient was diagnosed with bipolar disorder by a psychiatrist; (2) the patient felt that they were not in good shape with regard to bipolar disorder and wanted to know how to optimize the treatment based on the detailed examination during hospitalization; and (3) the attending physician could provide a referral letter to report the details of the current treatment. The staff verify whether these criteria are met by each applicant. Only eligible applicants who hoped to admit to the hospital under the given conditions were enrolled in the program (Fig. 1).

In this study, patients who entered the program between September 2020 and March 2022 were analyzed. This study was approved by the Juntendo University Ethics Committee for Medical Research (ID:M19-0278), and informed consent was obtained from all participants.

### 2.2. Diagnosis

A board-certified psychologist conducted a structured interview (SCID-5-RV: Structured Clinical Interview for DSM-5 Research Version)^[12]^ for each patient. After all the data were obtained, a conference was held with the attendant psychiatrists, a principal investigator (T.K.), a psychologist, and a psychiatric nurse whenever possible. These medical staff jointly discussed the Diagnostic and Statistical Manual of Mental Disorders, Fifth Edition diagnosis, clinical status, and current treatment based on the repeated clinical interviews, data of SCID-5-RV, and all other examinations, as well as observations of the patient’s behavior in the psychiatric ward by psychiatric nurses.

### 2.3. Examinations

Magnetic resonance imaging and cerebral blood flow scintigraphy using single-photon emission tomography (SPECT) with Tc-99mECD were performed for all patients. SPECT was analyzed by a radiologist, and statistical analysis images using easy Z-score imaging system were also used to rule out Alzheimer disease and Lewy body dementia^[13,14]^ The electroencephalography (EEG) by an international 10/20 method was performed to rule out epilepsy and other organic mental disorders^[15]^ and assessed by psychiatrists trained to assess EEG.

The following clinical assessments were performed by a psychiatrist: Hamilton Depression Rating Scale using a structured interview guide,^[16]^ Young Mania Rating Scale (Young et al, 1978), and the Frontal Assessment Battery.^[17]^ Patients completed a self-reported questionnaire, the Autism-Spectrum Quotient Japanese version (AQ-J).^[18]^ The Wechsler Adult Intelligence Scale-Fourth Edition^[19]^ was administered by a psychologist. Additional tests were conducted as required. The patient’s insight into the disorder was assessed through a conference based on clinical interviews and other information.

## 3. Results

A flowchart of the subject selection process is presented in Figure 1. We received inquiries from 224 candidates who were considering application to the program. Some patients decided not to be hospitalized because of various restrictions of the psychiatric ward, financial problems, schedule, and so on. Of these, 52 visited the outpatient clinic for the initial screening. Four patients excluded from the program were diagnosed with borderline personality disorder, and it was considered that hospitalization by another suitable framework would be better for these patients. The other 2 were in a full mood episode and were judged to be suitable for hospitalization for the treatment of the acute episode. Consequently, 48 patients entered the program and were admitted to the hospital. After admission, 5 of the 48 patients could not adjust to the environment of the psychiatric ward and were discharged before the completion of the program. The remaining 43 patients were included in the study.

Among them, 25 patients complained of self-perceived treatment-resistant depression, whereas 17 experienced self-perceived recurring episodes or persistent emotional instability. None of the patients had treatment-resistant manias. One patient was hospitalized for other reasons.

Of the 43 patients, 22 were men and 21 were women, with an age of 42.6 ± 13.3 years. The scores of symptom scales were Hamilton Depression Rating Scale = 7.6 ± 4.5, Young Mania Rating Scale = 2.3 ± 4.6, Frontal Assessment Battery = 17.0 ± 1.2, AQ-J = 22.3 ± 8.6, and full scale intelligence quotient = 101.9 ± 12.3.

### 3.1. Diagnosis

After performing the SCID-5-RV, we reviewed the diagnosis based on the Diagnostic and Statistical Manual of Mental Disorders, Fifth Edition based on various tests and medical history. Ten of the 43 patients were not diagnosed with bipolar disorder but with others, such as major depressive disorder. Remaining 33 were diagnosed with bipolar disorder. Detailed diagnoses are summarized in Table 1.

**Table 1:** Diagnosis reviewed at the 2-week inpatient program (n = 43).

Diagnosis	n	%
Non-bipolar	10	23.2
Major depressive disorder recurrent episode	5	11.6
Persistent depressive disorder (dysthymia)	1	2.3
Schizoaffective disorder bipolar type	2	4.6
Adjustment disorder	1	2.3
Substance-induced bipolar disorder	1	2.3
Bipolar	33	76.7
Bipolar I disorder	14	32.5
Bipolar II disorder	16	37.2
Other specified bipolar disorder	3	6.9

### 3.2. Organic factors

Some patients displayed notable findings on brain imaging and EEG. On brain magnetic resonance imaging (MRI), one patient was diagnosed with normal pressure hydrocephalus, one with cerebral infarction, and one with a prominent chronic ischemic lesion in the deep white matter suggestive of frontal lobe syndrome. All other MRI scans were considered to be within normal limits.

Cerebral blood flow scintigraphy by SPECT showed no clinically significant abnormal findings, except for 3 patients who had brain lesions revealed by MRI, as noted above. Five patients had mild hypoperfusion, suggestive of Alzheimer disease or Lewy body dementia with easy Z-score imaging system. However, dementias were ruled out by clinical evaluation, including additional assessment with the revised Hasegawa Dementia Scale and Mini Mental State Examination. Some of the other patients showed mild scattered hypoperfusion, mainly in the frontal region, but these findings were compatible with bipolar disorder.

None of the patients showed epileptic abnormalities in EEG.

### 3.3. Comorbidity

Eleven of the patients in this study had psychiatric comorbidities (Table 2). For those suspected to have developmental disorders by AQ-J and clinical interviews, the Parent Interview Rating Scale for Autism Spectrum Disorders, Text-Revised, Conners’ Adult ADHD Rating Scales, and the Autism Diagnostic Observation Schedule Second Edition were administered. Five were diagnosed with developmental disorders, 4 with autism spectrum disorder (ASD), and one with attention deficit hyperactivity disorder (AD/HD).

**Table 2:** Psychiatric comorbidities of the patients in this study (n = 43).

Diagnosis	n	%
ASD	4	13.2
Subthreshold ASD	4	13.2
ADHD	1	2.3
Subthreshold ADHD	2	4.6
Panic disorder	2	4.6
Bulimia nervosa	1	2.3
Anorexia nervosa/bulimia nervosa	1	2.3
OCD	1	2.3
Subthreshold borderline personality disorder	2	4.6
Total	18	

*Note*: Overlapping is present.

SCID-5-RV showed that 7 patients had other comorbidities as described in the Table 2.

### 3.4. Treatment

Among the 33 patients who were diagnosed with bipolar disorder, 7 did not receive treatment as recommended by the treatment guidelines for bipolar disorder of the Japanese Society of Mood Disorders.^[20]^ Three patients were not taking lithium without good reasons, 2 were taking 2 selective serotonin reuptake inhibitors, one was taking a tricyclic antidepressant, one was taking a noradrenalin reuptake inhibitor, and one was taking multiple drugs in the same classes at high doses (Table 3).

**Table 3:** Treatment received by patients diagnosed with bipolar disorder (n = 33).

Treatment	n	%
Compliant with the guidelines*	26	78.8
Deviated from the guidelines*	7	21.2
No use of Li	3	9.1
Improper use of antidepressants	4	12.1
Polypharmacy of drugs in the same classes	1	3.0

* Treatment guidelines for bipolar disorder by the Japanese Society of Mood Disorders.

### 3.5. Insight

Insufficient insight directly led to apparent treatment resistance in 11 of 43 patients (25.6%). Nine patients did not adhere to the treatment because of a lack of insight into the disease. Two patients complained that their bipolar disorder was unstable, but in reality, they suffered from psychological problems unrelated to mood symptoms.

### 3.6. Summary

Factors associated with self-perceived treatment resistant bipolar disorder among the 43 patients are summarized in Table 4.

**Table 4:** Causes of self-perceived treatment resistant bipolar disorder in the participants (n = 43).

Factors	n	%
Not diagnosed as bipolar disorder	10	23.2
Organic factors	3	7.0
Comorbid mental disorders	11	25.6
Did not receive adequate treatment	7	16.4
Nonadherence to the treatment because of a lack of insight	9	20.9
Attributing psychological problems to bipolar disorder	2	4.6
Genuine treatment resistant bipolar disorder	1	2.3
Total	43	

## 4. Discussion

In this study, 43 participants who regarded themselves as having uncontrolled bipolar disorder were examined in detail. Surprisingly, only one patient was diagnosed as having genuine treatment-resistant bipolar disorder without comorbidity, that is, persistent relapses of mood episodes of rapid cycling, despite adequate treatment. In addition, 10 (23%) patients were not diagnosed with bipolar disorder despite the fact that these patients self-reported that they had treatment-resistant bipolar disorder.

Among other patients who were diagnosed with bipolar disorder, the majority felt that their bipolar disorder was not well controlled because of symptoms unrelated to bipolar disorder, such as comorbid general medical or psychiatric conditions (14, 32%) or psychological problems (2, 5%).

Other patients did not receive adequate treatment because the attending physician did not follow the treatment guidelines (7, 16%), or the patients did not adhere to the treatment because of a lack of insight into the disease (9, 21%). These findings suggest that more education is needed to public and providing more appropriate services for diagnosis and treatment of bipolar disorder are necessary in the community. They also suggest that clinicians should be aware that genuine treatment resistance is rare in real-world clinical settings, and before labeling a patient with bipolar disorder as treatment-resistant, all clinical issues such as diagnosis, organic factors, comorbidities, treatment, and adherence should be carefully checked.

### 4.1. Diagnosis

The 9 patients who were not diagnosed with bipolar disorder did not have any detectable (hypo)manic episodes or had only drug-induced elation of mood. In these cases, the possibility that they would develop (hypo)manic episodes in the future could not be ruled out^[21]^ because approximately 20% of patients initially diagnosed with depression are later diagnosed with bipolar disorder.^[22,23]^ One patient was diagnosed with schizoaffective disorder, bipolar type. Compared with bipolar disorder, the prognosis of schizoaffective disorder, bipolar type is generally worse.^[24]^

### 4.2. Organic factors

Contrary to our expectation that organic factors or neurological conditions are a major determinant of treatment resistance in bipolar disorder, only 3 of 42 patients had such organic factors: normal pressure hydrocephalus and cerebrovascular diseases. In these cases, cognitive decline due to brain disease was considered a symptom of depressive episodes of bipolar disorder by the attending physicians as well as the patients. Although the frequency of such cases is not high, ruling out organic factors is necessary for a correct diagnosis.

### 4.3. Comorbidities

Five patients had a comorbid diagnosis of developmental disorder (ASD: 4; ADHD: 1). The comorbidity of ADHD in bipolar disorder has been reported to be approximately 20%^[25,26]^ and approximately 20% to 30% of patients with bipolar disorder meet the criteria for ASD.^[27,28]^ Although we did not consider these patients to have genuine treatment-resistant bipolar disorder in this study, the comorbidity of developmental disorders has been regarded as one of the factors causing treatment resistance in bipolar disorder.^[29,30]^ Thus, it might be reasonable to consider these 5 patients to have genuine treatment-resistant bipolar disorder. However, these patients did not have intractable mood symptoms but rather had difficulty in social life associated with the behavioral characteristics of ASD or AD/HD. One patient with AD/HD had hyperactivity and impaired impulse control during remission, but the patient thought that they had hypomanic symptoms. Patients with ASD had difficulties with social cognition and interpersonal relationships, and they felt that they were symptoms of bipolar disorder.

Patients with bipolar disorder have a high rate of comorbidity with ASD and AD/HD, partly due to overlapping genetic factors^[31–33]^ and a differential diagnosis and assessment of comorbidity is important.^[34,35]^ In the case of bipolar disorder with comorbid developmental disorders, in addition to the maintenance treatment of bipolar disorder, it is important to provide care and education tailored to developmental disorders.

The remaining 7 patients with comorbidities had anxiety disorders, eating disorders, obsessive compulsive disorder, or subthreshold borderline personality disorder. These patients felt that their mental status was not well maintained because of the symptoms of these comorbid mental disorders. Although we excluded these patients from genuine treatment-resistant bipolar disorder group, they did indeed suffer from mental symptoms despite adequate treatment for bipolar disorder. In this study, we did not conclude whether treatment for these comorbid mental disorders was adequate. It is possible that these patients did not receive adequate treatment for these comorbid mental disorders. The comorbidity of these mental disorders has been regarded as a factor for treatment refractoriness in bipolar disorder.^[36]^ It is necessary to treat comorbid mental disorders in conjunction with bipolar disorder.

### 4.4. Treatment

Seven patients were diagnosed with bipolar disorder but did not receive treatment as recommended by the treatment guidelines. Three patients were not treated with lithium, the first-choice drug, without any good reason. Other patients were treated with antidepressants, including tricyclic antidepressants, which are not recommended by the Japanese Society of Mood Disorders guidelines and might have caused mood instability. One patient with comorbid AD/HD was treated with a noradrenalin reuptake inhibitor, and it is possible that this medication, together with AD/HD, might have caused mood instability. The treatment of comorbid AD/HD in bipolar disorder is a matter of debate because psychostimulants and other drugs for AD/HD potentially worsen the course of bipolar disorder. Treatment options should be optimized on a patient-by-patient basis.

### 4.5. Adherence

Lack of insight is associated with poor adherence to treatment in bipolar disorder.^[37]^ Thus, to achieve full insight, psychoeducation is provided in clinical practice.^[38]^ However, psychoeducation does not always improve the insight in patients with bipolar disorder.^[39]^ In this study, 9 patients did not adhere to the treatment because of a lack of insight or other psychological issues. One patient misidentified her euthymic state as depressive by idealizing the hypomanic state. One patient did not accept any standard treatment and claimed side effects. One patient repeatedly relapsed, and this was associated with a premature return to work at partial remission. One patient did not follow the recommendations for lifestyle, such as maintaining a schedule. Although we did not classify these cases as receiving inadequate treatment, more extensive psychoeducation might improve insight or understanding of the disease and the treatment.

## 5. Limitations and conclusions

This study has several limitations. The number of participants was small. The selection bias of this study hampers the generalization of the findings. No validated assessment tools were used to assess insight and adherence. We could not try adequate treatment and verify recovery in the patients receiving inadequate treatment because of the limited period of hospitalization. Thus, some patients who had not received adequate treatment at the time of assessment might have suffered from genuine treatment-resistant bipolar disorder after adequate treatment.

Despite these limitations, this study sheds light on the factors associated with apparent treatment resistance in bipolar disorder. Most patients with apparent treatment-resistant bipolar disorder are not regarded as having genuine treatment-resistant bipolar disorder, and there is room for improvement after assessing these factors and providing adequate management for these issues. This study also highlights the difficulty of public education and providing adequate services for bipolar disorder in the community, and emphasizes their importance.

## Author contributions

**Conceptualization:** Tadafumi Kato.

**Data curation:** Tadafumi Kato, Fujimura Toshimasa.

**Formal analysis:** Tadafumi Kato, Fujimura Toshimasa.

**Funding acquisition:** Tadafumi Kato.

**Investigation:** Tadafumi Kato, Fujimura Toshimasa, Daiki Taira.

**Project administration:** Tadafumi Kato.

**Resources:** Toshimasa Fujimura, Yoshihiro Uchida, Keitaro Takahashi, Kanako Yamasuji, Kentaro Shimizu, Yasuhito Nagai, Naoto Yoshinari, Tomoe Hirata, Kazuma Fujimoto, Yui Kurosawa, Seita Yasuda, Akane Yoshikawa, Yoshihide Takeshita, Masanobu Ito, Chihiro Kakiuchi.

**Supervision:** Tadafumi Kato.

**Writing – review & editing:** Tadafumi Kato

## References

[1] Miller S Dell’Osso B Ketter TA. The prevalence and burden of bipolar depression. J Affect Disord. 2014;169:S3–11. 25533912 10.1016/S0165-0327(14)70003-5

[2] Kanba S Kato T Terao T. Guideline for treatment of bipolar disorder by the Japanese Society of Mood Disorders, 2012. Psychiatry Clin Neurosci. 2013;67:285–300. 23773266 10.1111/pcn.12060

[3] Yatham LN Kennedy SH Parikh SV. Canadian Network for Mood and Anxiety Treatments (CANMAT) and International Society for Bipolar Disorders (ISBD) 2018 guidelines for the management of patients with bipolar disorder. Bipolar Disord. 2018;20:97–170. 29536616 10.1111/bdi.12609 PMC5947163

[4] Malhi GS Bell E Bassett D. The 2020 Royal Australian and New Zealand College of Psychiatrists clinical practice guidelines for mood disorders. Aust N Z J Psychiatry. 2021;55:7–117. 33353391 10.1177/0004867420979353

[5] Radua J Grunze H Amann BL. Meta-analysis of the risk of subsequent mood episodes in bipolar disorder. Psychother Psychosom. 2017;86:90–8. 28183076 10.1159/000449417

[6] Solomon DA Keitner GI Miller IW. Course of illness and maintenance treatments for patients with bipolar disorder. J Clin Psychiatry. 1995;56:5–13. 7836345

[7] Bennabi D Charpeaud T Yrondi A. Clinical guidelines for the management of treatment-resistant depression: French recommendations from experts, the French Association for Biological Psychiatry and Neuropsychopharmacology and the fondation FondaMental. BMC Psychiatry. 2019;19:262. 31455302 10.1186/s12888-019-2237-x PMC6712810

[8] Parker GB Graham RK. Clinical characteristics associated with treatment-resistant bipolar disorder. J Nerv Ment Dis. 2017;205:188–91. 27105455 10.1097/NMD.0000000000000517

[9] Tohen M Frank E Bowden CL. The International Society for Bipolar Disorders (ISBD) Task Force report on the nomenclature of course and outcome in bipolar disorders. Bipolar Disord. 2009;11:453–73. 19624385 10.1111/j.1399-5618.2009.00726.x

[10] Fountoulakis KN Yatham LN Grunze H. The CINP guidelines on the definition and evidence-based interventions for treatment-resistant bipolar disorder. Int J Neuropsychopharmacol. 2020;23:230–56. 31802122 10.1093/ijnp/pyz064 PMC7177170

[11] Hidalgo-Mazzei D Berk M Cipriani A. Treatment-resistant and multi-therapy-resistant criteria for bipolar depression: consensus definition. Br J Psychiatry. 2019;214:27–35. 30520709 10.1192/bjp.2018.257 PMC7613090

[12] First MB Williams JBW Karg RS. Structured Clinical Interview for DSM-5 - Research Version. Arlington, VA: American Psychiatric Association; 2015.

[13] Matsuda H. [Neurological diseases and SPECT--analysis using easy Z-score imaging system (eZIS)]. Brain Nerve. 2007;59:487–93. 17533974

[14] Imabayashi E Soma T Sone D. Validation of the cingulate island sign with optimized ratios for discriminating dementia with Lewy bodies from Alzheimer’s disease using brain perfusion SPECT. Ann Nucl Med. 2017;31:536–43. 28547521 10.1007/s12149-017-1181-4 PMC5517560

[15] Klem GH Luders HO Jasper HH. The ten-twenty electrode system of the International Federation The International Federation of Clinical Neurophysiology. Electroencephalogr Clin Neurophysiol Suppl. 1999;52:3–6. 10590970

[16] Williams JB. A structured interview guide for the Hamilton Depression Rating Scale. Arch Gen Psychiatry. 1988;45:742–7. 3395203 10.1001/archpsyc.1988.01800320058007

[17] Dubois B Slachevsky A Litvan I. The FAB: a Frontal Assessment Battery at bedside. Neurology. 2000;55:1621–6. 11113214 10.1212/wnl.55.11.1621

[18] Wakabayashi A Tojo Y Baron-Cohen S. [The Autism-Spectrum Quotient (AQ) Japanese version: evidence from high-functioning clinical group and normal adults]. Shinrigaku Kenkyu. 2004;75:78–84. 15724518 10.4992/jjpsy.75.78

[19] Drozdick LW Raiford SE Wahlstrom D. The Wechsler Adult Intelligence Scale—Fourth Edition and the Wechsler Memory Scale—Fourth Edition. Contemporary intellectual assessment: Theories, tests, and issues, 4th ed. New York, NY, US: The Guilford Press. 2018:486–511.

[20] Kanba S Kato T Terao T. Guideline for treatment of bipolar disorder by the Japanese Society of Mood Disorders, 2012. Psychiatry Clin Neurosci. 2013;67:285–300. 23773266 10.1111/pcn.12060

[21] Takeshima M Oka T. Association between the so-called “activation syndrome” and bipolar II disorder, a related disorder, and bipolar suggestive features in outpatients with depression. J Affect Disord. 2013;151:196–202. 23790740 10.1016/j.jad.2013.05.077

[22] Akiskal HS Walker P Puzantian VR. Bipolar outcome in the course of depressive illness phenomenologic, familial, and pharmacologic predictors. J Affect Disord. 1983;5:115–28. 6222091 10.1016/0165-0327(83)90004-6

[23] Hu YH Chen K Chang IC. Critical predictors for the early detection of conversion from unipolar major depressive disorder to bipolar disorder: nationwide population-based retrospective cohort study. JMIR Med Inform. 2020;8:e14278. 32242821 10.2196/14278 PMC7165312

[24] Harrow M Grossman LS Herbener ES. Ten-year outcome: patients with schizoaffective disorders, schizophrenia, affective disorders and mood-incongruent psychotic symptoms. Br J Psychiatry. 2000;177:421–6. 11059995 10.1192/bjp.177.5.421

[25] Merikangas KR Jin R He JP. Prevalence and correlates of bipolar spectrum disorder in the world mental health survey initiative. Arch Gen Psychiatry. 2011;68:241–51. 21383262 10.1001/archgenpsychiatry.2011.12 PMC3486639

[26] Schiweck C Arteaga-Henriquez G Aichholzer M. Comorbidity of ADHD and adult bipolar disorder: a systematic review and meta-analysis. Neurosci Biobehav Rev. 2021;124:100–23. 33515607 10.1016/j.neubiorev.2021.01.017

[27] Skokauskas N Frodl T. Overlap between autism spectrum disorder and bipolar affective disorder. Psychopathology. 2015;48:209–16. 26278909 10.1159/000435787

[28] Joshi G Biederman J Petty C. Examining the comorbidity of bipolar disorder and autism spectrum disorders: a large controlled analysis of phenotypic and familial correlates in a referred population of youth with bipolar I disorder with and without autism spectrum disorders. J Clin Psychiatry. 2013;74:578–86. 23842009 10.4088/JCP.12m07392

[29] Strakowski SM. CANMAT and ISBD 2018 guidelines for the management of patients with bipolar disorder. Bipolar Disord. 2018;20:393–4. 29676513 10.1111/bdi.12650

[30] Espluga-Frigola N Cardoner N Pamias-Massana M. Comorbidity of autism spectrum disorder and bipolar disorder. Actas Esp Psiquiatr. 2017;45:79–88. 28353295

[31] Asherson P Young AH Eich-Hochli D. Differential diagnosis, comorbidity, and treatment of attention-deficit/hyperactivity disorder in relation to bipolar disorder or borderline personality disorder in adults. Curr Med Res Opin. 2014;30:1657–72. 24804976 10.1185/03007995.2014.915800

[32] Naguy A. ADHD-juvenile bipolar disorder: mimics and chameleons! World J Pediatr. 2018;14:525–7. 29446039 10.1007/s12519-018-0124-z

[33] Mazefsky CA Oswald DP Day TN. ASD, a psychiatric disorder, or both? Psychiatric diagnoses in adolescents with high-functioning ASD. J Clin Child Adolesc Psychol. 2012;41:516–23. 22642847 10.1080/15374416.2012.686102 PMC3601822

[34] Brainstorm C Anttila V Bulik-Sullivan B. Analysis of shared heritability in common disorders of the brain. Science. 2018;360:eaap8757. 29930110 10.1126/science.aap8757 PMC6097237

[35] O’Connell KS McGregor NW Lochner C. The genetic architecture of schizophrenia, bipolar disorder, obsessive-compulsive disorder and autism spectrum disorder. Mol Cell Neurosci. 2018;88:300–7. 29505902 10.1016/j.mcn.2018.02.010

[36] Sharma V Mazmanian D Persad E. A comparison of comorbid patterns in treatment-resistant unipolar and bipolar depression. Can J Psychiatry. 1995;40:270–4. 7553547 10.1177/070674379504000509

[37] Gutierrez-Rojas L Martinez-Ortega JM Perez-Costillas L. Illness insight and medication adherence among patients with bipolar disorder. J Nerv Ment Dis. 2020;208:481–7. 32040060 10.1097/NMD.0000000000001151

[38] Stafford N Colom F. Purpose and effectiveness of psychoeducation in patients with bipolar disorder in a bipolar clinic setting. Acta Psychiatr Scand Suppl. 2013;127(Suppl. 442):11–8. 10.1111/acps.12118 23581788

[39] Casellas E Raventos B Pineiro-Rios M. A real-world study of the association between a brief group psychoeducation and the course of bipolar disorder. Int J Environ Res Public Health. 2021;18:5019. 34068535 10.3390/ijerph18095019 PMC8126006

